# Early Nontraumatic Periprosthetic Femoral Fracture After Total Hip Arthroplasty: A Case Report

**DOI:** 10.1155/cro/1962733

**Published:** 2026-09-15

**Authors:** Yuta Kataoka, Kenichiro Doi, Koichi Kinoshita, Taiki Matsunaga, Masahiro Suzuki, Yoshiaki Hideshima, Fumihiro Yoshimura, Yoshitomo Oya, Takuaki Yamamoto

**Affiliations:** ^1^ Department of Orthopedic Surgery, Fukuoka University Faculty of Medicine, Fukuoka, Japan, fukuoka-u.ac.jp

**Keywords:** case report, finite element analysis, periprosthetic femoral fracture, total hip arthroplasty

## Abstract

This report describes a rare case of early nontraumatic periprosthetic femoral fracture following total hip arthroplasty. The patient was a 74‐year‐old woman with osteoarthritis of the hip who underwent left total hip arthroplasty via a posterior approach using a tapered, rectangular, cementless femoral stem. The patient progressed well through early rehabilitation and was ambulating independently by postoperative Day 8. However, on postoperative Day 9, she experienced sudden‐onset thigh pain and was unable to bear weight while ascending stairs. Radiographs demonstrated a short oblique fracture of the left femur that was located distal to the stem tip and classified as a Vancouver type C (postoperative) fracture. Open reduction and internal fixation were performed using a locking plate in conjunction with cables and screws. At the 2‐year follow‐up, the patient had regained her ability to ambulate independently, and radiographs confirmed successful bone union. Finite element analysis suggested that concentration of stress around the tip of the stem as a result of flexed stem alignment, together with stair climbing, may have contributed to the periprosthetic femoral fracture. Caution is warranted when inserting a femoral stem in a flexed position because of an increased risk of periprosthetic femoral fracture.

## 1. Introduction

The incidence of total hip arthroplasty (THA) has risen in recent years, driven by broader surgical indications and ongoing advances in prosthetic implant design. In parallel with this trend, the rate of complications associated with THA, particularly periprosthetic femoral fractures (PFFs), has also increased [1–3]. PFFs have been reported to occur in 0.1%–27.8% of intraoperative cases and 0.07%–18% of postoperative cases [4]. Established risk factors include advanced age, female sex, osteoporosis, and low‐energy trauma [5].

The majority of PFFs are observed within the first year after THA and are often attributed to inadequate osteointegration of cementless femoral stems [5]. Some studies define PFFs that arise within 90 days postoperatively as “early PFF” [6]. There have also been sporadic reports of nontraumatic PFFs that occurred several years after THA [7–9]. Therefore, nontraumatic PFFs that occur during the early postoperative period are thought to be rare. This report presents a case of nontraumatic PFF that developed during the early postoperative phase following THA.

This case report was prepared in accordance with the CARE guidelines. The CARE Checklist is provided as Data S1.

## 2. Case Presentation

A 74‐year‐old woman presented to our hospital with pain in both hips and was subsequently diagnosed with bilateral osteoarthritis of the hip. She underwent right THA first because the pain was more severe on the right side. Her pain improved after surgery; she was able to walk independently, and her postoperative course was favorable. A contralateral THA was scheduled 1 year after the initial procedure. The patient had been taking oral minodronate at 50 mg every 4 weeks for 1 year following her right THA as treatment for osteoporosis. There was no relevant family history.

**Figure 1:**
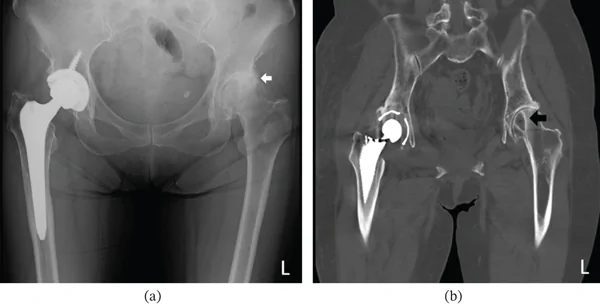
Imaging findings for a 74‐year‐old woman who presented with progressive left thigh pain. (a) Anteroposterior radiograph of the pelvis demonstrating subtle joint space narrowing and osteophyte formation at the femoral head, with no evidence of cortical thickening or beaking in the left femur (white arrow). (b) Coronal computed tomography image showing bone cysts in the left femoral head (black arrow).

At the time of preoperative evaluation for the left THA, although the patient was able to ambulate independently without a cane, she reported intermittent claudication localized to the left side. Supine range of motion of the left hip was as follows: flexion, 110°; extension, 0°; abduction, 20°; adduction, 5°; external rotation, 10°; and internal rotation, 0°. The Japanese Orthopedic Association Hip‐Disease Evaluation Questionnaire [10] score was 61 points, and the Harris Hip Score [11] was 60 points. Bone mineral density at the left femoral neck was 82% (T‐score−1.3) of the young adult mean. Preoperative imaging demonstrated severe osteoarthritis in the left hip (Figure 1a,b).

**Figure 2:**
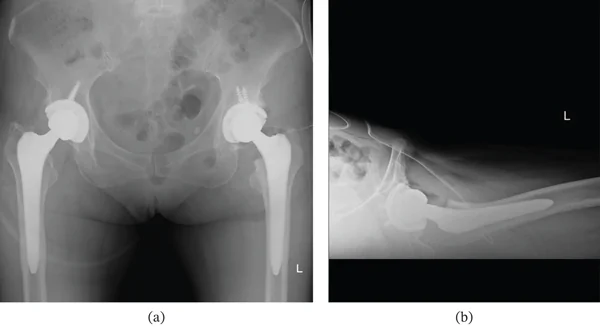
Findings on routine postoperative images obtained to assess for intraoperative and postoperative fractures as well as cup positioning after total hip arthroplasty. (a) Anteroposterior radiograph showing no evidence of stem subsidence or periprosthetic fracture. (b) Cross‐table lateral radiograph showing no evidence of stem subsidence or periprosthetic fracture.

**Figure 3:**
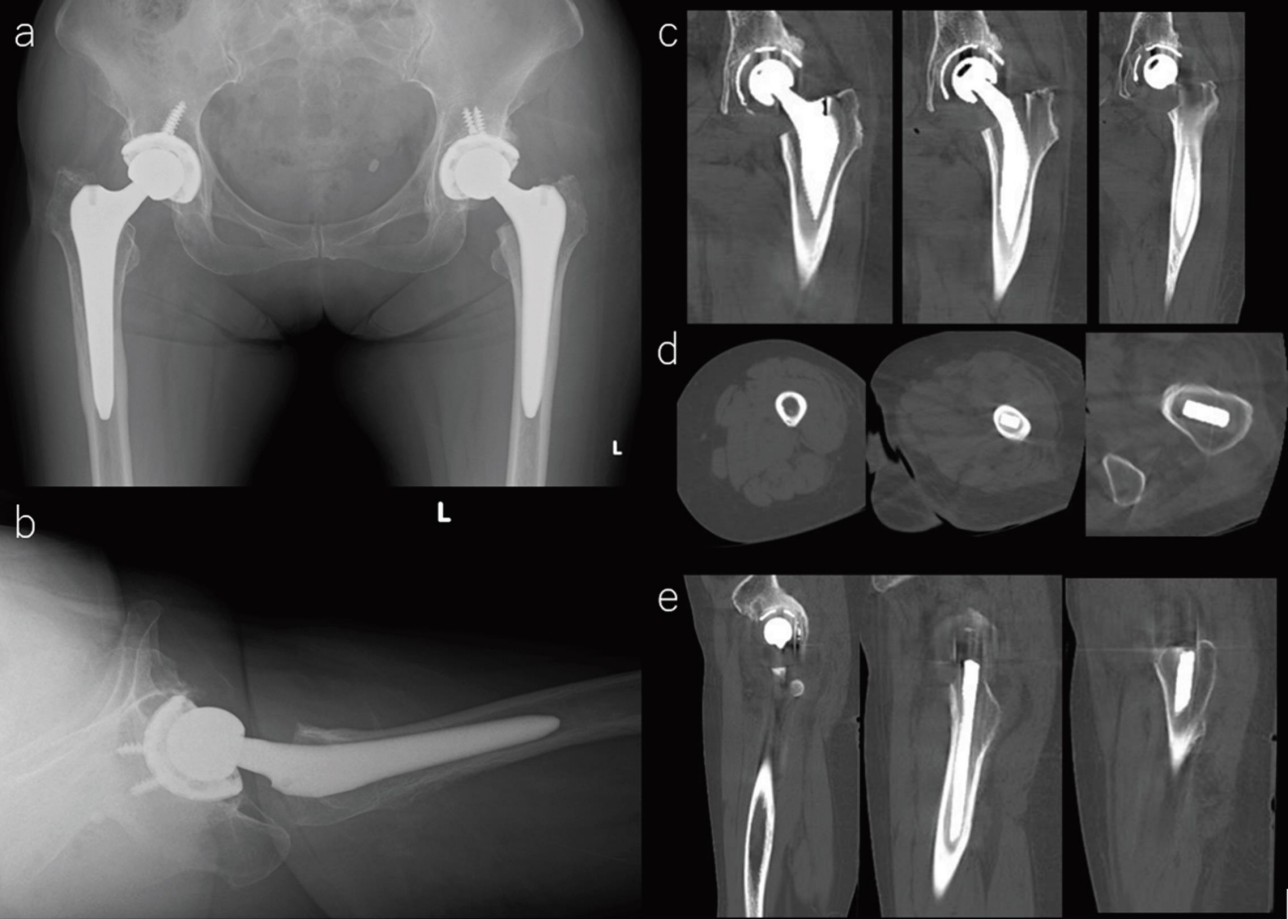
Routine radiographs and computed tomography scans obtained on Day 7 after total hip arthroplasty. No evidence of stem subsidence or periprosthetic fracture was seen on (a) an anteroposterior radiograph, (b) a cross‐table lateral radiograph, or (c) a coronal, (d) axial, or (e) sagittal computed tomography scan.

Left THA was performed using a posterior approach with cementless components, namely, a SQRUM acetabular shell (Kyocera, Kyoto, Japan) and an Inheritor femoral stem (Kyocera) (Figure 2a,b). On the lateral radiograph, the femoral stem was inserted with 3° of flexion (Figure 2b). A woodpecker device was not used during the procedure. No intraoperative or immediate postoperative periprosthetic fractures were identified radiographically (Figure 2a,b). There was also no evidence of periprosthetic fracture or stem subsidence on radiographs or computed tomography (CT) scans obtained on postoperative Day 7 as part of routine imaging for detection of postoperative fractures and assessment of the positioning of the cup (Figure 3a–e). Sagittal stem alignment remained unchanged, measuring 3.37° of flexion immediately after surgery and 3.32° of flexion on postoperative Day 7. The patient was allowed to resume full weight‐bearing ambulation with a walker on postoperative Day 2 and transitioned to unassisted ambulation by postoperative Day 8.

**Figure 4:**
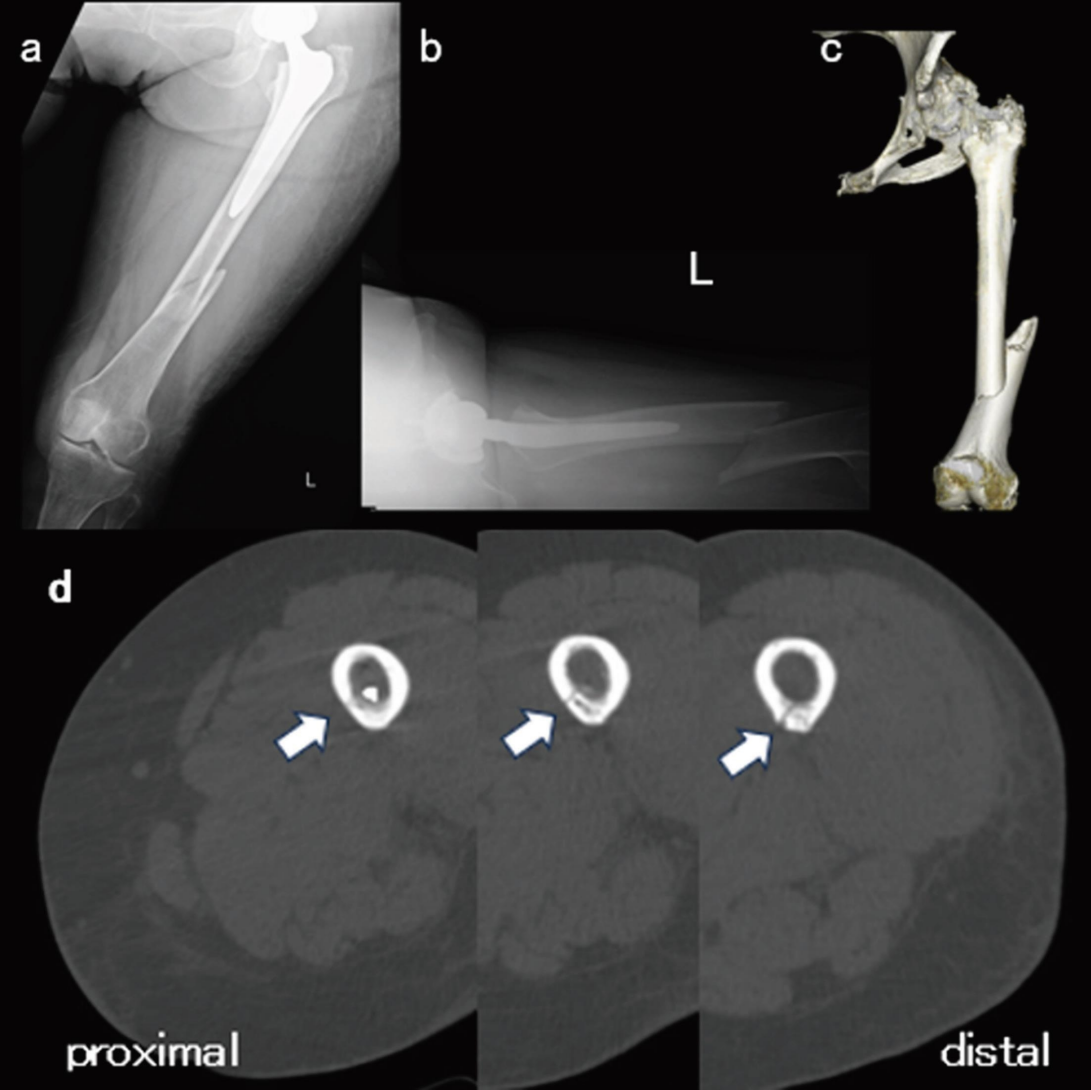
Images obtained 9 days after total hip arthroplasty when the patient developed acute severe pain in the left thigh when climbing stairs. (a) Anteroposterior radiograph showing an oblique periprosthetic femoral fracture distal to the femoral stem, which was classified as Vancouver type C (postoperative). (b) Cross‐table lateral radiograph confirming fracture displacement and cortical disruption. (c) Three‐dimensional computed tomography reconstruction image demonstrating an oblique Vancouver type C (postoperative) fracture without evidence of implant loosening. (d) Axial computed tomography images showing a fracture posterior to the femur at the level of the distal tip of the stem. The white arrows indicate the fracture site.

**Figure 5:**
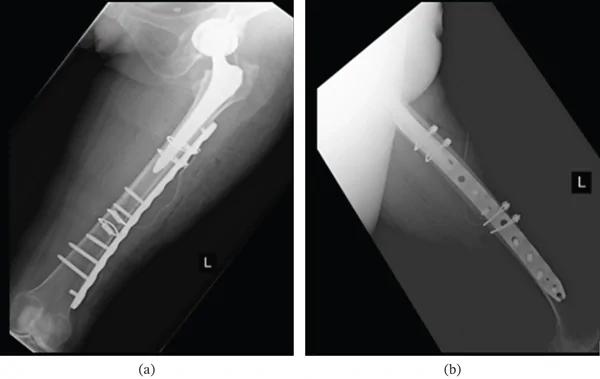
Radiographs obtained following internal fixation of a periprosthetic femoral fracture. (a) Oblique radiograph showing open reduction and internal fixation using a long locking plate and cerclage cables spanning the femoral shaft. (b) Lateral radiograph confirming appropriate alignment and implant positioning without evidence of hardware failure.

However, on postoperative Day 9, the patient experienced acute, severe pain in the left thigh for the first time during stair‐climbing exercises, which made standing difficult. Imaging revealed a short oblique PFF distal to the femoral stem (Figure 4a–d). At the time of fracture, sagittal stem alignment measured 3.34° of flexion, whereas stem subsidence was 6.07 mm. A diagnosis of PFF was made, and the patient underwent open reduction and internal fixation 2 days later (Figure 5a,b). Surgical management involved a 6‐cm lateral incision to expose and reduce the fracture, which was initially stabilized with two cerclage cables. Additional 6‐ and 3‐cm lateral incisions were made over the distal stem and femoral shaft, respectively. A 12‐hole curved femoral shaft locking plate (NCB, Zimmer, Warsaw, Indiana, United States) was applied to span at least 6 cm distal to the stem. Seven locking screws were inserted, and the cerclage cables were secured to the plate via attachment devices passed through the additional incisions.

Unrestricted range of motion was permitted from postoperative Day 1. Partial weight‐bearing ambulation with a walker was started at 4 weeks, and full weight‐bearing ambulation was allowed at 6 weeks. Adjuvant therapies included initiation of low‐intensity pulsed ultrasound and an oral human parathyroid hormone preparation starting on postoperative Day 2. Follow‐up radiographs at 6 weeks did not show any evidence of implant loosening, fracture displacement, or other abnormalities. The patient resumed independent ambulation and was discharged home on postoperative day 54. At the 2‐year follow‐up, the patient was ambulating without claudication, and radiographic imaging confirmed complete bone union at the fracture site. A structured patient timeline of the patient′s diagnostic, surgical, and therapeutic management is presented in Table 1.

**Table 1:** Timeline of clinical events.

Time point	Clinical events
Preoperative	Diagnosed with left hip OA and scheduled for THA
POD 0	Left THA performed
POD 8	Independent ambulation achieved
POD 9	Sudden thigh pain while climbing stairs; Vancouver type C PFF diagnosed
POD 11	ORIF with locking plate, cables, and screws
2 years	Bone union confirmed; independent ambulation regained

We speculated that insertion of the stem in a flexed position during THA may have contributed to the PFF that occurred in this case. Accordingly, a finite element analysis was performed to evaluate the changes in stress on the femur after THA.

## 3. Finite Element Analysis

### 3.1. Methods

#### 3.1.1. Image Processing and Coordinate System

The three‐dimensional geometry of the femur was reconstructed from preoperative CT DICOM data with a slice thickness of 0.5 mm. The femoral region was segmented by image processing to create three‐dimensional reconstruction images. An anatomical coordinate system was defined for the femur using the anterior–posterior, medial–lateral, and proximal–distal directions as reference axes. The angle of insertion of the femoral stem in the sagittal plane was defined relative to this coordinate system.

#### 3.1.2. Construction of the Finite Element Model

A three‐dimensional finite element model was constructed using Mechanical Finder Version 13 based on the reconstructed femoral geometry. The femoral neck was resected to simulate the postoperative conditions before insertion of the stem.

#### 3.1.3. Material and Contact Properties

The cancellous bone and the inner cortical bone were meshed using first‐order tetrahedral elements with an edge length of 1.5 mm. Material properties were assigned as heterogeneous materials based on CT values using the density–elastic modulus conversion formula described by Keyak et al. [12], with a Poisson′s ratio of 0.4. The outer cortical bone was modeled using three‐layer shell elements (thickness, 0.001 mm), and the material properties were set to be identical to those of the adjacent solid elements. The contact condition between the femoral stem and femur was defined using a friction coefficient of 0.4, consistent with previous reports [13, 14].

#### 3.1.4. Virtual Insertion of the Femoral Stem

A three‐dimensional STL (Standard Triangle Language) model of a size 3 high‐offset femoral stem (INHERITOR, Kyocera, Kyoto, Japan), corresponding to the implant used in this patient was used. The stem was inserted from the resected surface of the femoral neck toward the femoral shaft and was positioned at a height that did not cause leg length discrepancy. Varus–valgus alignment was set to 0° in the coronal plane and femoral anteversion to 25°. In the sagittal plane, two insertion conditions were evaluated: neutral alignment and 3° of flexion alignment. The 3° of flexion alignment condition was selected to represent a clinically relevant deviation from neutral alignment in accordance with a previous report [15].

#### 3.1.5. Loading and Boundary Conditions

Natural physiological loading was approximated by using the loading conditions for scenarios involving standing and climbing stairs. For standing, a joint reaction force of 2400 N was applied to the femoral head at an angle of 15° proximal medial relative to the femoral axis, and an abductor muscle force of 1200 N was applied to the greater trochanter at an angle of 20° distal lateral relative to the femoral axis [16, 17]. A load of 2400 N was applied from anterior to posterior at the center of the femoral head to simulate the loading pattern when climbing stairs. The patient′s body weight was approximately 50 kg (corresponding to a force of approximately 490 N). Therefore, the applied load of 2400 N corresponded to approximately 4.9 times body weight. This loading magnitude was selected based on previous biomechanical research demonstrating that reaction forces at the hip joint may reach 5–6 times body weight when climbing stairs [18]. Furthermore, in vivo measurements have reported mean peak forces of approximately 1900 N and peak forces of up to 4200 N at the hip joint when climbing stairs [19]. Therefore, the load applied was considered physiologically relevant. The boundary conditions were set for full restraint at the femoral condyles.

#### 3.1.6. Outcome Measures and Data Analysis

**Figure 6:**
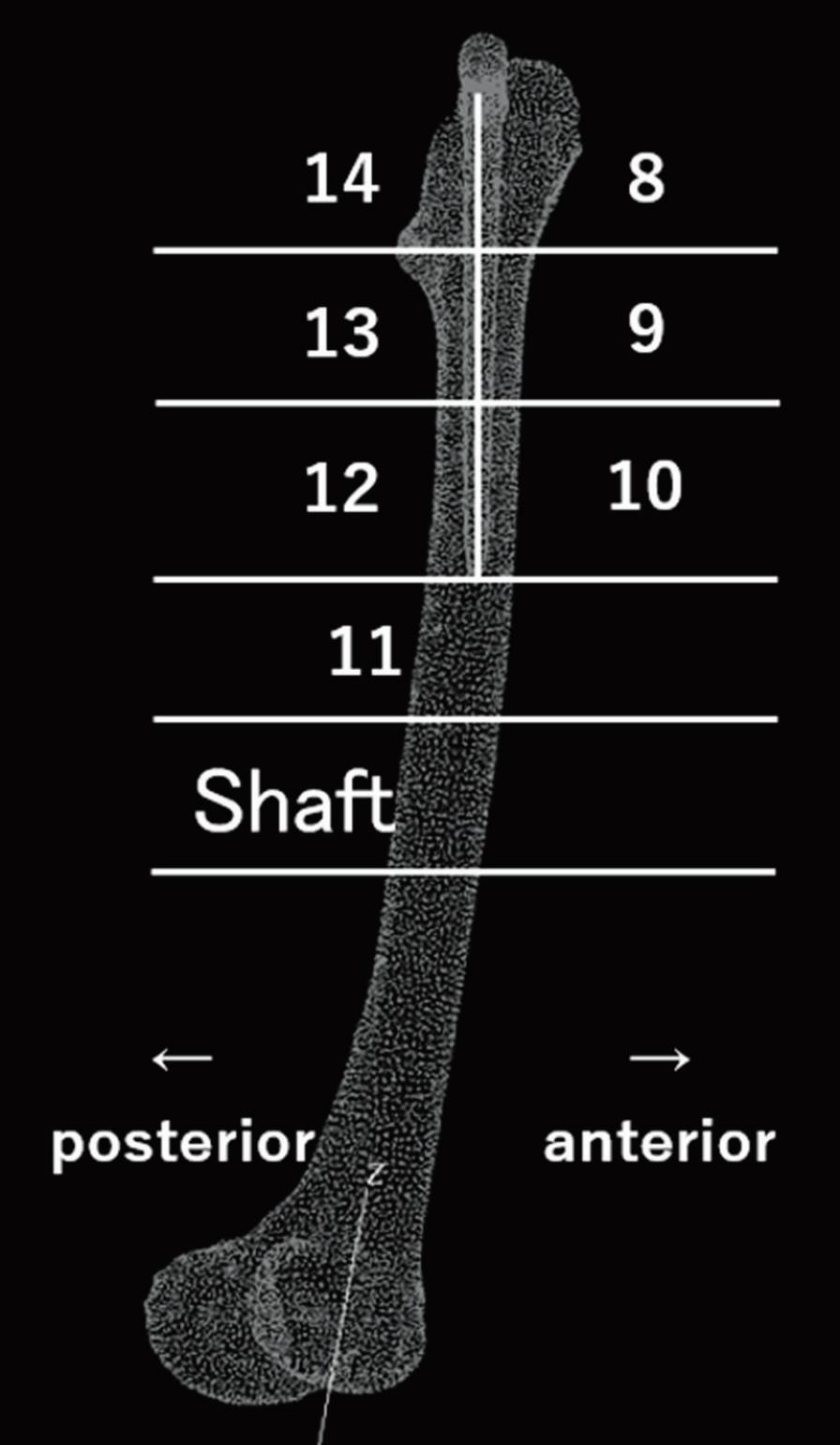
Stress was assessed in the sagittal plane by finite element modelling across Gruen Zones 8–14 and the femoral shaft.

The primary outcome was mean von Mises stress. Analyses were performed for neutral alignment and for alignment in 3° of flexion under both loading conditions. The regions of interest were newly defined in the sagittal plane according to the Gruen classification and were termed sagittal Gruen Zones 8–14 and the shaft (Figure 6).

## 4. Results

**Figure 7:**
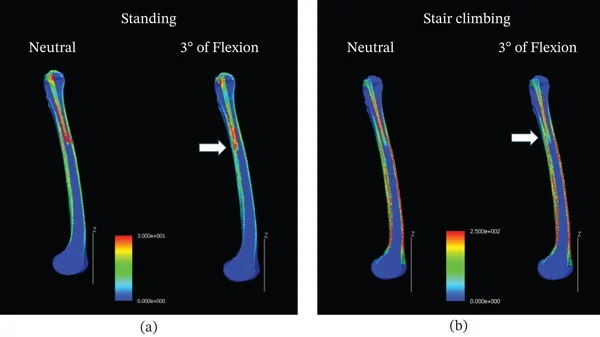
Finite element analysis showed increased stress on the femur after left total hip arthroplasty using a size 3 stem with high offset. Under both (a) standing and (b) stair climbing conditions, von Mises stress at the posterior distal femur around the stem was greater with 3° of stem flexion than with neutral alignment (white arrows).

**Figure 8:**
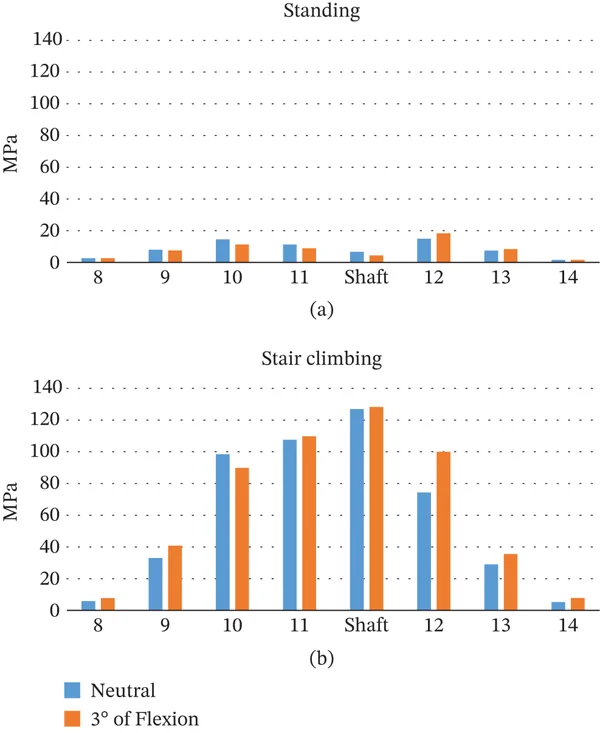
Distributions of von Mises stress in the sagittal plane determined by finite element modeling when the stem is inserted in the neutral position and with 3° of flexion. (a) Standing condition. (b) Stair climbing condition.

Finite element analysis revealed increased stress in Gruen Zone 12 during both standing and climbing stairs when the stem was inserted in 3° of flexion compared with neutral alignment under both standing and stair‐climbing conditions (Figure 7a,b and 8a,b, Table 2). Specifically, the von Mises stress in sagittal Gruen Zone 12 increased from 15.65 MPa to 19.18 MPa (22.56% increase) under the standing condition and from 74.61 MPa to 99.73 MPa (33.66% increase) under the stair climbing condition. Climbing stairs also markedly increased stress overall.

**Table 2:** Representative von Mises stress values (MPa) and percentage changes in each sagittal Gruen zone under both (a) standing and (b) stair climbing conditions.

**(a) Standing**								

	8	9	10	11	Shaft	12	13	14

Neutral (MPa)	3.3	9.08	15.61	11.43	7.92	15.65	7.94	2.51
3°of flexion (MPa)	3.23	7.57	12.19	8.89	5.34	19.18	9.02	2.48
Percentage change (%)	−1.9	−16.7	‐21.92	−22.2	−32.63	22.56	13.66	−1.01

**(b) Stair climbing**								

	8	9	10	11	Shaft	12	13	14

Neutral (MPa)	5.9	32.64	98.26	107.26	126.46	74.61	27.99	6
3°of flexion (MPa)	7.64	40.48	89.85	109.24	127.52	99.73	35.19	6.87
Percentage change (%)	29.36	24.02	−8.56	1.84	0.84	33.66	25.72	14.6

*Note:* Percentage change (%) was calculated as [(3° of Flexion–Neutral alignment)/Neutral alignment] × 100. Positive values indicate an increase, and negative values indicate a decrease. Von Mises stress values are presented as rounded values, whereas percentage changes were calculated from the corresponding unrounded values and then rounded for presentation.

## 5. Discussion

Numerous risk factors for postoperative PFF have been reported, including advanced age, female sex, traumatic osteoarthritis, osteoporosis, rheumatoid arthritis, proximal femoral deformities, previous surgery on the affected hip, use of cementless stems and press‐fit fixation, cortical perforation by the implant, presence of cortical stress risers, low‐energy trauma, peri‐implant osteolysis, implant loosening, occult fractures, and previous joint replacement surgery [1, 2, 4, 20–23]. Our patient had several potential risk factors for PFF, including advanced age, female sex, and a history of treatment for osteoporosis. Use of a cementless femoral stem further aligns with the established risk factors. There was no evidence of periprosthetic fracture intraoperative or immediate postoperative radiograph, or on the radiographs and CT images obtained 7 days after surgery. The patient remained capable of full weight‐bearing without thigh pain until postoperative Day 8. This clinical course supports the exclusion of an occult fracture during the early postoperative period.

In this case, the femoral stem implanted during THA was inserted with 3° of flexion. CT imaging at the time of the fracture demonstrated a fracture line extending from the tip of the stem to the distal femur. Although there have been no previous reports indicating that insertion of a stem in a flexed position increases the risk of periprosthetic fracture, we considered this possibility. Accordingly, we performed a finite element analysis to evaluate the changes in stress on the femur. This analysis revealed that stress in Gruen Zone 12, where the tip of the stem was in contact with the cortical bone, was greater when the stem was inserted in 3° of flexion than when it was in neutral alignment when standing and climbing stairs. Furthermore, climbing stairs markedly increased stress overall, with particularly prominent increases in stress in the distal stem region (Gruen Zones 10–12, femoral shaft) (Figure 8a,b). CT images obtained at the time of fracture demonstrated that the fracture line originated at the tip of the stem and extended distally, whereas no fracture was identified in the proximal femur around the stem. These imaging findings were consistent with the stress concentration around the tip of the stem observed in the finite element analysis.

Based on these findings, we believe that, in this case, insertion of the stem in a flexed position caused contact between the tip of the stem and the cortical bone, increasing stress at the contact site and triggering the PFF. The increased stress on the distal stem when climbing stairs likely contributed to the fracture.

Our finite element analysis had some limitations. First, it was based on simulation and did not fully replicate in vivo conditions, including the effects of soft tissues and muscle forces, which may influence transmission of load and distribution of stress in the femur. Second, the stair climbing condition was simulated using simplified loading patterns and boundary conditions. Therefore, it may not have fully captured the complex and dynamic in vivo loading environment that occurs when ascending stairs in reality, such as time‐dependent changes in muscle activation and joint kinematics. Third, secondary osteoporosis and other metabolic bone disorders were not specifically investigated in this patient. Additionally, patient‐specific factors, including bone quality and other unrecognized biomechanical and biological factors that may influence fracture susceptibility, were not fully evaluated. Therefore, the fracture mechanism should be interpreted as multifactorial rather than attributable solely to stem alignment. Finally, the finite element analysis was based on the stem alignment observed on the postoperative Day 7 CT. Although no apparent sagittal stem alignment change was identified between the immediate postoperative and postoperative Day 7 imaging, no imaging was available immediately before the fracture. Quantitative radiographic assessment demonstrated 6.07 mm of stem subsidence at the time of fracture, whereas sagittal stem alignment remained essentially unchanged (3.34° of flexion) compared with the immediate postoperative period (3.37°) and postoperative Day 7 (3.32°). Therefore, the radiographic change at the time of fracture was considered to mainly reflect stem subsidence rather than a change in sagittal alignment. However, because no imaging was available immediately before the fracture, the exact timing of stem subsidence and the possibility of subtle changes in stem alignment immediately before the fracture could not be determined. This uncertainty should be considered when interpreting the biomechanical findings.

This case suggests that changes in stress induced by insertion of the stem in a flexed position may contribute to periprosthetic fractures after THA. The results of our finite element analysis suggest that concentration of stress around the tip of the stem as a result of flexed alignment of the stem may have contributed to the PFF in this case. The fracture line identified on CT originated from the stem tip and extended distally, supporting the finite element finding that stress was concentrated in this region. However, these findings should be interpreted with caution because the reasons for the fracture were likely to be multifactorial and cannot be attributed to stem flexion alone. Given that this analysis represents only an adjunctive evaluation based on a single case and cannot establish causality, further large‐scale and systematic studies are needed. From a clinical standpoint, achieving appropriate stem alignment during THA may be considered to optimize biomechanical conditions and potentially reduce the risk of such complications.

## Funding

No funding was received for this manuscript.

## Ethics Statement

The patient was informed that her data could be submitted for publication

## Consent

Informed consent for publication of this case was obtained from the patient.

## Conflicts of Interest

The authors declare no conflicts of interest.

## Supporting information


**Supporting Information** Additional supporting information can be found online in the Supporting Information section. Data S1: CARE Checklist for the case report.

## Data Availability

The data that support the findings of this study are available from the corresponding author upon reasonable request.
